# Guiding Cochlear Implant Electrode Insertion Using Impedance Measurements: A Computational Simulation Study

**DOI:** 10.3390/s26216870

**Published:** 2026-10-30

**Authors:** Enver Salkim, Andreas Demosthenous

**Affiliations:** 1 Department of Electronic and Automation, Muş Alparslan University, 49100 Muş, Türkiye; 2 Department of Electronic and Electrical Engineering, University College London, London WC1E 7JE, UK; a.demosthenous@ucl.ac.uk

**Keywords:** bio-impedance analysis, boundary proximity sensing, cochlear implant (CI), finite element modeling, real-time trajectory tracking, sensor-guided surgery

## Abstract

Intraoperative tissue trauma during cochlear implant (CI) insertion remains a primary cause of residual hearing loss. Because insertion currently relies largely on clinician experience without direct visualization within the optically obscured inner ear, real-time guidance is critical for minimizing mechanical contact with delicate anatomical structures. This study evaluates multi-channel electrical impedance for real-time trajectory tracking and boundary proximity detection during automated electrode insertion. A computational finite-element model of the human cochlea was used to analyze complex impedance profiles across eight bipolar configurations (E1–E2 to E15–E16) and 25 insertion models (M1–M25), yielding 2592 evaluations across central, medial, and lateral trajectories under varying bulk tissue conductivities. At 1kHz, reactive contributions were negligible (X<25 Ω, ϕ≤0.27°), confirming an overwhelmingly resistive response (R>2400 Ω, >99.5% of total impedance). Bulk conductivity scaled baseline impedance magnitudes without substantially altering the spatial profile geometry. Across the three insertion trajectories, impedance exhibited a characteristic U-shaped evolution, initially decreasing as the electrode array (EA) advanced toward the scala tympani (ST) center before increasing near the terminal insertion region. Boundary proximity further increased impedance variation, with mean impedance values increasing by 24–38% for the medial trajectory and 25–35% for the lateral trajectory relative to the middle baseline. Localized impedance increases were observed for the medial pair E3–E4 (65% at M20) and the lateral pair E1–E2 (88% at M23). The differential impedance profiles indicate that boundary-induced proximity effects substantially exceed baseline spatial variations, demonstrating the sensitivity of multi-channel impedance measurements to electrode–wall proximity. These findings support the potential use of impedance signatures as a real-time feedback signal for boundary proximity detection, clinician guidance, and closed-loop trajectory adjustment during automated CI insertion. Recognizing the characteristic U-shaped impedance profile and its sensitivity to boundary proximity may provide valuable intraoperative feedback for monitoring electrode-array position and reducing the risk of boundary contact and associated mechanical trauma.

## 1. Introduction

The cochlea plays a critical role in the auditory system by converting acoustic signals into neural impulses that are transmitted to the brain for sound perception [1]. Damage to cochlear hair cells or dysfunction of the auditory nerve can result in sensorineural hearing loss, one of the most prevalent sensory disorders worldwide [1]. Globally, more than 5% of the population, comprising approximately 432 million adults and 34 million children, experiences disabling hearing loss, rendering it a major public health concern [2]. Beyond its clinical consequences, unaddressed hearing impairment leads to profound communication barriers, social isolation, reduced educational attainment, and restricted employment opportunities, imposing substantial socioeconomic burdens. The global annual cost of unaddressed hearing loss is estimated to approach US$1 trillion [2]. For individuals with severe to profound sensorineural hearing loss, cochlear implants (CIs) represent the standard-of-care intervention. By restoring auditory access, CIs significantly enhance communication capabilities, improve overall quality of life, and mitigate the long-term socioeconomic impacts of hearing impairment [3,4].

**Figure 1:**
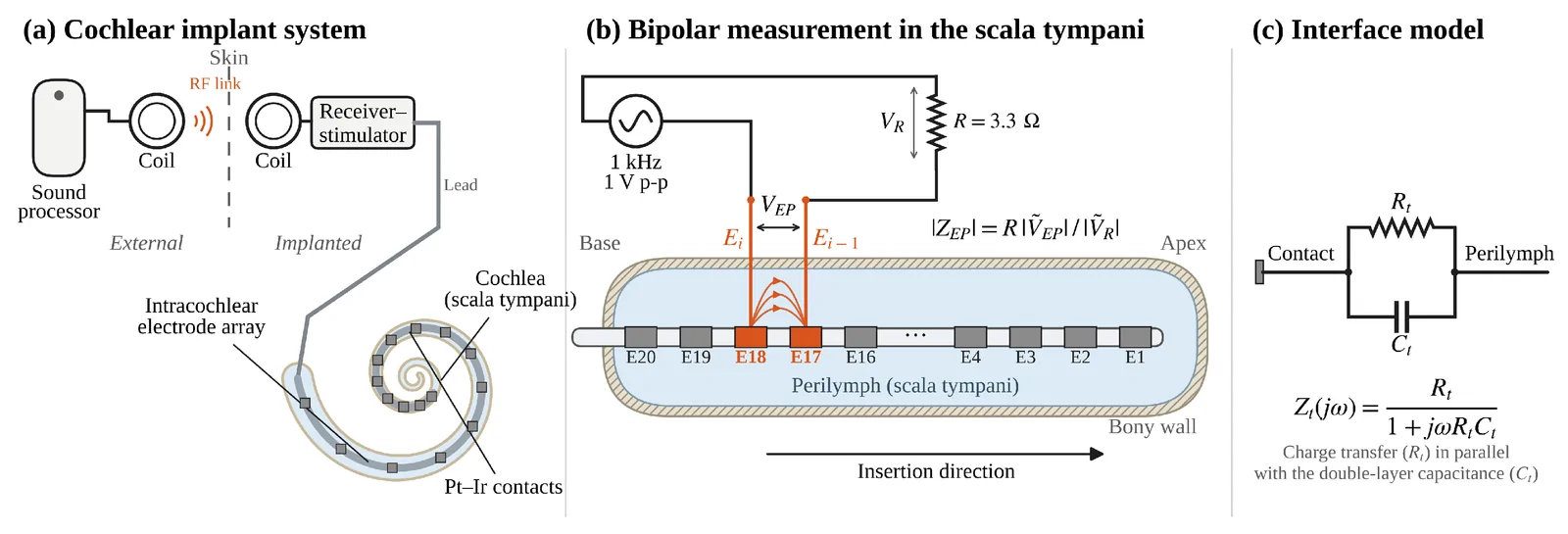
Overview of a cochlear implant and the impedance sensing configuration. (**a**) Main components of a cochlear implant system: the external sound processor transmits power and data across the skin via an RF coil link to the implanted receiver–stimulator, which drives the intracochlear electrode array through the lead. (**b**) Unrolled representation of the electrode array within the perilymph-filled scala tympani. A sinusoidal voltage (1 kHz, 1 V peak to peak) is applied in series with a sense resistor *R* across an adjacent contact pair ($E_{i}$ and $E_{i-1}$), and the impedance is obtained from the inter-electrode voltage $V_{EP}$ and the resistor voltage $V_{R}$. (**c**) Equivalent circuit of the electrode–tissue interface, consisting of the charge-transfer resistance $R_{t}$ in parallel with the double-layer capacitance $C_{t}$.

The CI is a neural prosthetic device engineered to bypass non-functional or damaged hair cells by delivering direct electrical stimulation to surviving spiral ganglion neurons and auditory nerve fibers via an electrode array (EA) positioned within the scala tympani (ST) [5,6]. The main components of a conventional CI system are illustrated in Figure 1a, depicting the external sound processor and implanted receiver–stimulator that work synchronously to capture, encode, and transmit electrical stimuli. Although cochlear implantation is a mature, safe, and routine surgical procedure, surgical outcomes are highly sensitive to implant technology, electrode array design, and insertion mechanics [5,7]. Recent clinical evidence indicates that atraumatic and precisely controlled insertion techniques, including image-guided and robot-assisted approaches, markedly reduce intracochlear trauma and preserve delicate cochlear structures, thereby optimizing postoperative speech perception and residual hearing preservation [5,8,9].

Because conventional surgical electrode insertion relies predominantly on passive tactile feedback, procedure-related complications, such as array translocation, tip fold-over, incomplete insertion, or basilar membrane disruption, frequently occur [5,7,10]. Accurate intra-scalar positioning is essential for maximizing auditory performance and preserving residual low-frequency hearing [10]. Off-axis placement or trajectory deviations elevate mechanical contact forces against the outer cochlear wall, basilar membrane, or osseous spiral lamina, thereby compounding structural trauma [11,12]. Anatomical variations in ST dimensions and curvature further complicate manual insertion dynamics [13]. Previous studies have shown that consistent, atraumatic placement confined entirely within the ST is strongly correlated with optimal post-implant speech outcomes [14,15]. Consequently, maintaining real-time awareness of electrode position during insertion is paramount for minimizing mechanical trauma [10,16].

To mitigate insertion-induced trauma, modern developments have yielded flexible pre-curved arrays, perimodiolar designs, and active robotic deployment systems [5,8]. Despite these technological advances, surgeons still lack real-time intraoperative visualization regarding intra-scalar array positioning during deployment. To address this limitation, several intraoperative monitoring modalities have been investigated, including intraoperative computed tomography (CT), force sensing, electrocochleography (ECochG), electric field imaging (EFI), and impedance-based tracking [10,16,17,18]. While intraoperative CT provides definitive structural visualization, its routine adoption is limited by ionizing radiation exposure, high equipment costs, and surgical workflow disruptions [9]. Consequently, non-invasive, implant-based intraoperative monitoring techniques have gained considerable traction. In particular, electrical impedance measurement has emerged as a promising, real-time feedback mechanism capable of detecting wall proximity, identifying trauma events, and guiding placement without supplemental imaging modalities [10,19].

The CI simulation and impedance recording setup is shown in Figure 1b. Electrical impedance monitoring relies on the fundamental conductivity contrast within the inner ear: high-conductivity perilymph fluid offers lower electrical resistance compared to the surrounding resistive bony walls and delicate soft tissue structures [20,21,22]. Consequently, an electrode approaching an intra-cochlear boundary demonstrates distinct impedance profile shifts relative to one positioned within the central lumen [22]. Impedance signatures can thus provide immediate spatial feedback to guide array positioning, avoid mechanical contact, and minimize intracochlear trauma [20,21,23]. However, evaluating these electrical phenomena in vivo is severely constrained by the deep temporal bone embedding of the cochlea, anatomical variability across subjects, and physical limitations in experimentally measuring localized intra-scalar current distributions [24,25]. Finite element method (FEM) computational models overcome these experimental barriers by providing a controlled, high-resolution platform to simulate and characterize intra-cochlear electric field behavior [10,16,26,27].

**Figure 2:**
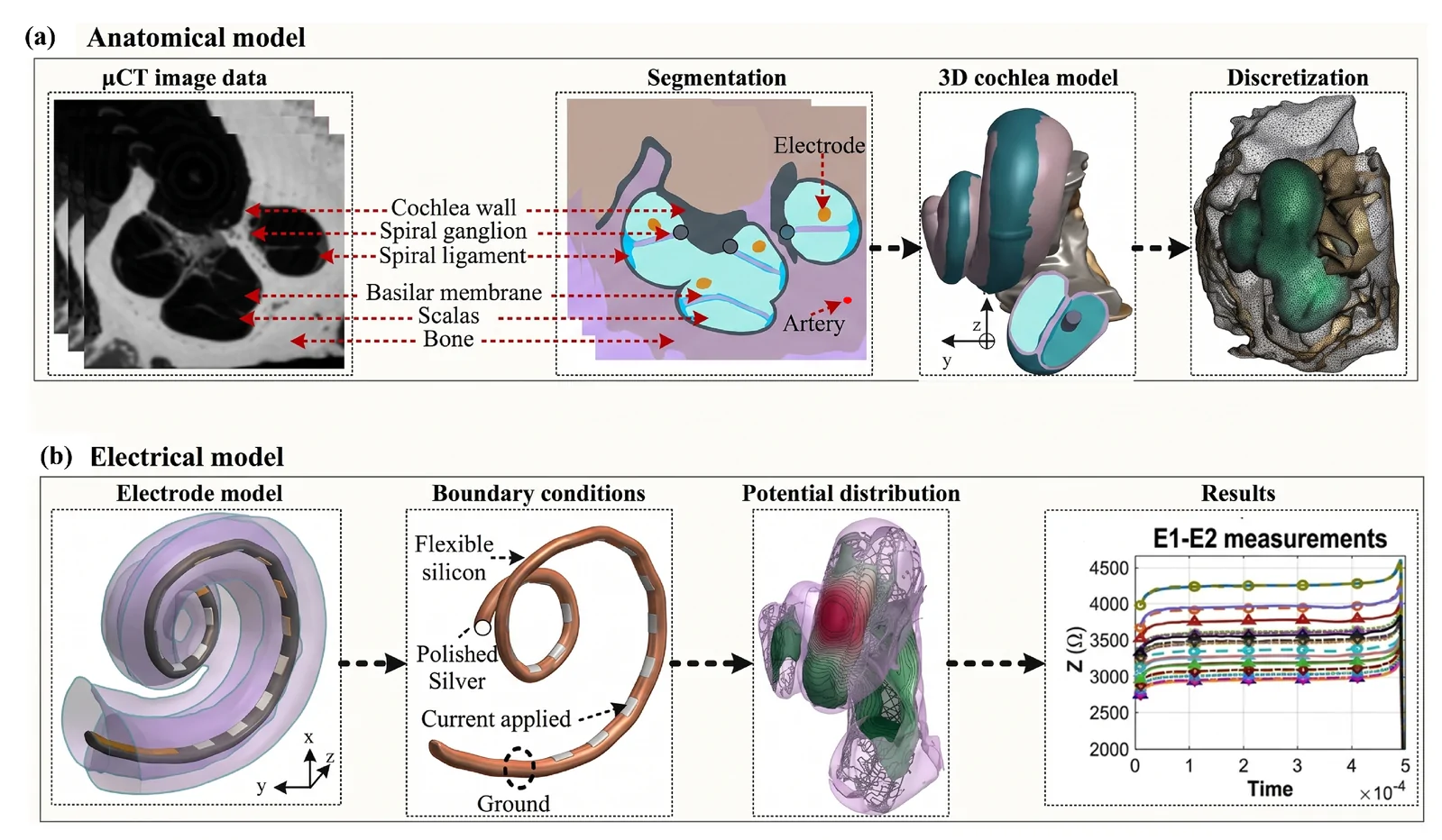
Workflow for human cochlear modeling and electrical potential simulation. (**a**) Anatomical structures of the cochlea are segmented from imaging data. A three-dimensional finite element (FE) model is reconstructed from the segmented images using labeling and surface-smoothing techniques in ScanIP. (**b**) A cochlear implant electrode array is incorporated into the 3D cochlear model, followed by mesh generation and the application of electrical current with appropriate boundary conditions. The finite element analysis computes the resulting electrical potential distribution throughout the cochlear volume, enabling the evaluation of impedance variations.

**Figure 3:**
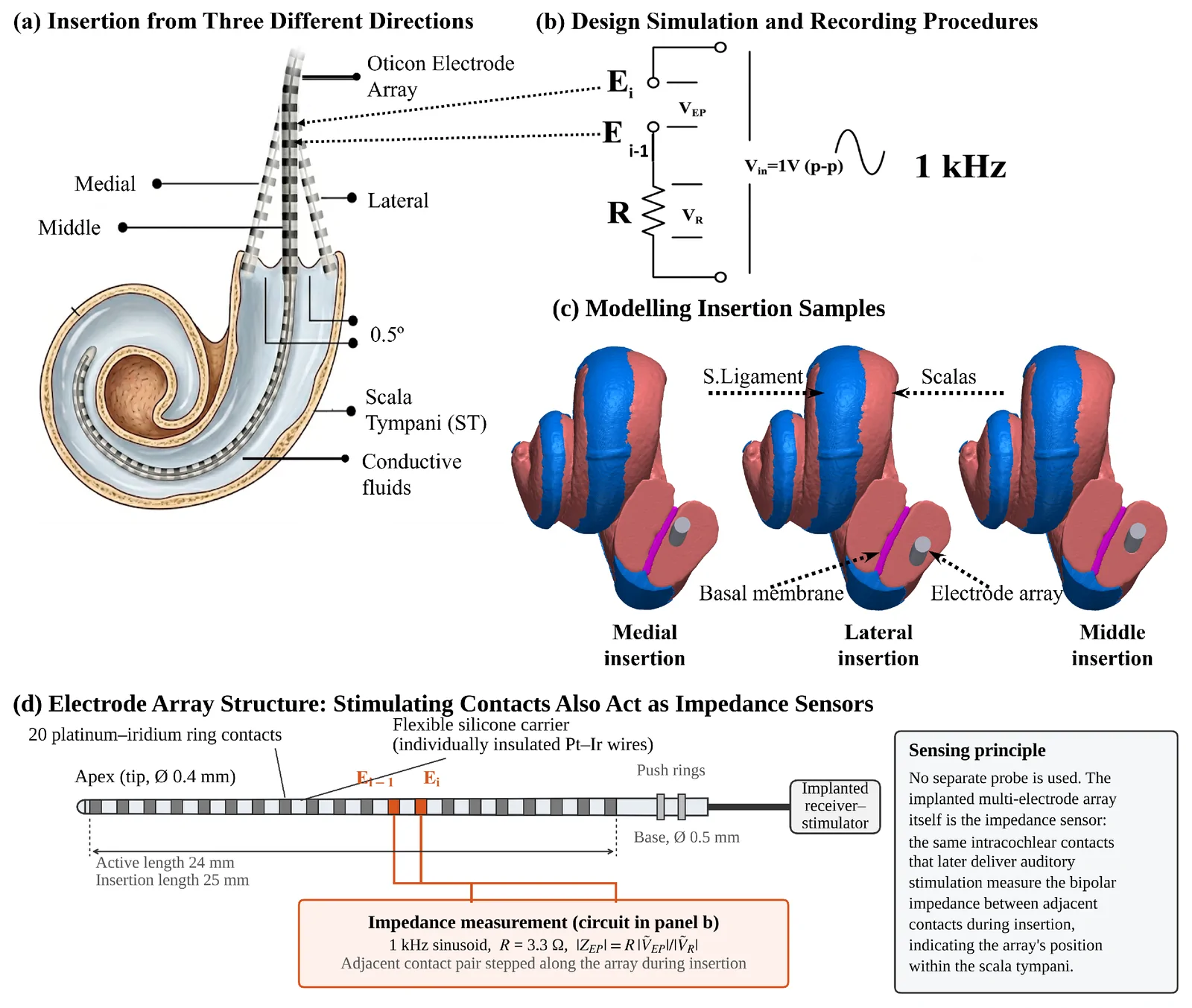
(**a**) Schematic of the three electrode array (EA) insertion trajectories considered in this study: medial, middle, and lateral. (**b**) Measurement circuit: a sinusoidal voltage ($V_{in}=1\;V$ peak to peak, 1kHz) is applied across an adjacent pair of contacts in series with a sense resistor R=3.3Ω; the inter-electrode voltage $V_{EP}$ and the resistor voltage $V_{R}$ are recorded to compute the impedance. (**c**) EA insertions along the medial, lateral, and middle trajectories in an anatomically realistic model of the human cochlea. (**d**) Structure of the EA: 20 platinum–iridium ring contacts on a flexible silicone carrier (active length 24 mm, insertion length 25 mm, diameter 0.4 mm at the apex and 0.5 mm at the base). The intracochlear contacts used for stimulation also act as impedance sensors: the impedance is measured between an adjacent contact pair ($E_{i-1}$, $E_{i}$) using the circuit in (**b**), and the pair is stepped along the array during insertion.

In this study, the relationship between electrode–ground impedance, bipolar channel impedance, intra-scalar proximity, and insertion depth were investigated using detailed FEM volume conductor modeling. A high–resolution multilayered three-dimensional (3D) anatomical model of the human cochlea was reconstructed from micro-computed tomography (μCT) datasets (Figure 2a,b). An electrode array based on the commercial Oticon EA (Oticon Medical AB, Askim, Sweden) design incorporating 20 platinum–iridium contacts was integrated into the ST geometry (Figure 1 and Figure 2). Array deployments along three distinct trajectories (medial, middle and lateral) were simulated throughout sequential insertion stages (Figure 3) to determine whether spatial impedance variations can serve as a robust, real-time diagnostic signal for intraoperative trajectory guidance.

The remainder of this paper is organized as follows. Section 2 describes the development of the 3D anatomical volume-conductor model derived from μCT data, the electrode-array geometry, tissue conductivity formulations, FEM simulation protocol, and impedance extraction methodology. Section 3 presents the simulation results, focusing on the relationship between impedance variation, electrode-array trajectory, boundary proximity, and insertion depth. Section 4 discusses the clinical and biomechanical implications of the findings and summarizes the main conclusions of the study.

## 2. Materials and Methods

### 2.1. Development of a 3D Model of the Cochlea

Accurate FEM simulations require a detailed 3D anatomical representation of the cochlea and its surrounding structures. In this study, the cochlear volume conductor and adjacent tissue layers were reconstructed from high-resolution μCT images of a human cochlea, acquired with a voxel resolution of 2.24 × 2.24 × 5 μm [10]. To reduce computational demands, the region of interest was limited to the cochlea and nearby anatomical structures. The image data set was subsequently resampled to an isotropic voxel size of 9.6 μm, resulting in a volume with dimensions of 930 × 930 × 1014 voxels.

The μCT dataset was imported into Simpleware ScanIP (Synopsys, Mountain View, CA, USA) for image processing and tissue segmentation. Segmentation involved identifying and grouping voxels corresponding to specific anatomical structures, enabling the generation of 3D models of the cochlear tissues, as shown in Figure 2. The reconstructed volume conductor included the scala tympani, scala vestibuli, cochlear wall, basilar membrane, spiral ligament, stria vascularis, spiral ganglion, and associated vascular structures, as summarized in Table 1. The surrounding osseous tissue was defined as the outer bony layer of the model.

A combination of automated and manual segmentation techniques was employed to ensure anatomical accuracy and computational efficiency [28]. Image enhancement was performed using recursive Gaussian, median, and mean smoothing filters to improve tissue differentiation based on grayscale intensity values. Initial segmentation of individual tissue layers was carried out automatically using grayscale thresholding, while manual refinement was applied where necessary to correct morphology and eliminate artifacts. Morphological operations, including dilation, erosion, opening, and closing, were used to refine tissue boundaries and fill discontinuities. Finally, Boolean operations were applied to remove overlaps between adjacent structures and establish well-defined interfaces between tissue layers for FEM analysis.

### 2.2. Electrode Array (EA) Design and Insertion Procedures

The EA model was developed to simulate electrical stimulation within different regions of the cochlea, based on the commercially available EVO^®^ electrode array (Oticon Medical AB, Askim, Sweden). The device features a soft, straight design and was modeled with a 25 mm insertion depth and a 24 mm active stimulating length. The array consists of a flexible silicone carrier embedding 20 platinum–iridium microelectrodes, labeled sequentially from E1 (apical end) to E20 (basal end) as shown in Figure 2 and Figure 3.

The cochlear geometry was first reconstructed in ScanIP (Synopsys, Mountain View, USA) by manually defining the ST centerline. This was achieved by tracing the spiral trajectory of the cochlear duct and capturing its spatially varying cross-sectional shape. The resulting centerline coordinates were exported as *x*, *y*, and *z* data and imported into COMSOL Multiphysics^®^ 5.2a (COMSOL Ltd., Cambridge, UK). The EA geometry was then generated by interpolating these coordinates and sweeping the electrode structure along the reconstructed cochlear path. In the computational model, the thin electrode contacts were represented as boundary surfaces within the simulation domain.

**Figure 4:**
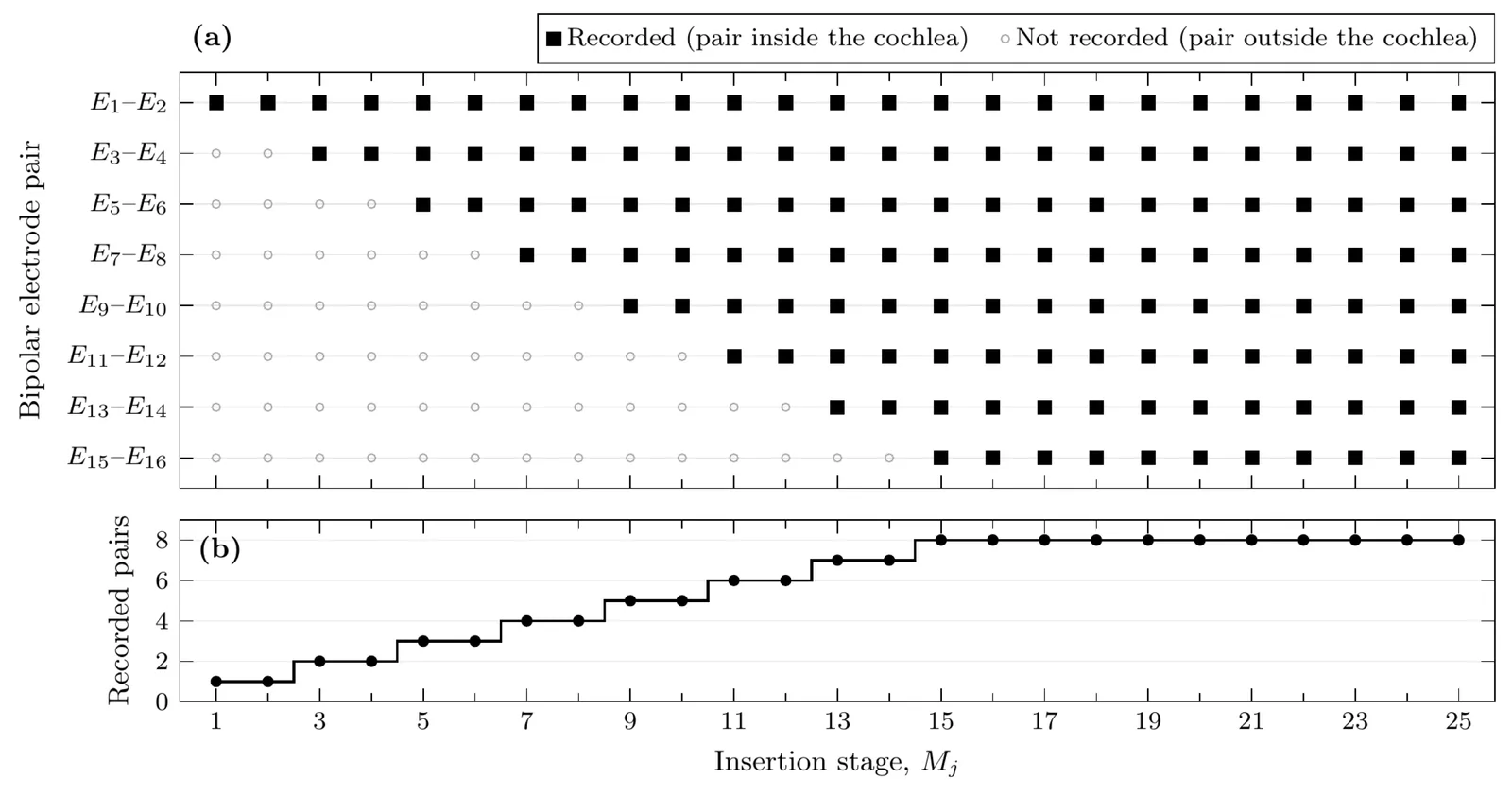
Bipolar impedance recording scheme during electrode array insertion. (**a**) Recording map of each adjacent contact pair $E_{2k-1}$–$E_{2k}$ (k=1,…,8) as a function of the insertion stage Mj (j=1,…,25). Filled squares mark the stages at which the pair lies within the cochlea and its impedance is recorded; open circles mark the stages before the pair has entered the cochlea. (**b**) Number of pairs recorded at each insertion stage, which increases from one pair at M1 to all eight pairs from M15 onwards.

To investigate the effect of insertion trajectory, three orientations were simulated: medial, medial, and lateral (Figure 3). The middle case was aligned with the geometric center of the ST, whereas the medial and lateral cases were obtained by applying an angular rotation of 0.5° about the reference axis toward the modiolus and the lateral wall, respectively. At the beginning of each simulation, the EA was placed outside the ST model, with only the first electrode pair (E1–E2) immersed in the conductive medium. Electrode pairs outside the ST exhibited high impedance values, representing an open-circuit condition. Consequently, these results were excluded from further analysis as the recording matrix shown in Figure 4.

The EA was then inserted into the ST along a 25 mm path at a constant rate using uniform displacement steps. During insertion, impedance measurements were recorded for eight electrode pairs (from E1–E2 through E15–E16) across all configurations. This resulted in 25 samples of measurement for the full insertion, corresponding to a spatial resolution of roughly one measurement per millimeter of advancement. To account for the variability in cochlear tissue conductivities reported across the existing literature [29], the conductivity of the ST was parameterized across six distinct levels ($\sigma_{1}–\sigma_{6}$) to evaluate the impact of the conductivity range on the outcome. Across all evaluated trajectories, this formulation yielded 144 unique insertion configurations as shown in Figure 4 (6STconductivitylevels×3insertiontrajectories). Impedance profiles were systematically recorded across bipolar electrode pairings, resulting in 2592 individual electrode-pair evaluations.

### 2.3. FEM Discretization and Simulation

The cochlear geometries were imported into COMSOL Multiphysics^®^ v5.2a for FEM analysis and discretized using tetrahedral elements to solve the governing partial differential equations. The simulations were executed on a 64-bit multi-core computing system using an iterative conjugate gradient solver. The overall solution accuracy was strongly dependent on mesh density and local element resolution.

To ensure accurate representation of the electrical field distribution, a non-uniform meshing strategy was employed. Highly refined tetrahedral elements were assigned to the scalae and surrounding soft tissue regions, where rapid spatial variations in potential were expected. In these areas, the minimum element size was on the order of 1 μm, with a controlled growth rate of 1.1 to preserve geometric fidelity. In contrast, regions farther from the electrode–tissue interface were discretized using progressively coarser elements (approximately 0.1–1 mm minimum size) to reduce computational cost without significantly affecting accuracy.

The electrode structures were meshed with adaptive refinement depending on the specific insertion configuration, ensuring appropriate resolution of the electrode–electrolyte interface. The outer bony structures, which are located distant from the region of interest, were modeled using relatively coarse tetrahedral meshes.

A mesh convergence assessment was performed to verify numerical stability, and the final model consisted of approximately 3–5 million elements depending on the configuration. This meshing strategy provided a balance between computational efficiency and accurate resolution of localized electrical field variations within the cochlea.

The resulting FEM framework enabled the evaluation of spatially dependent impedance variations as a function of electrode position and insertion orientation within the ST.

### 2.4. Transient Electrical Potential and Bioimpedance Modeling

Spatiotemporal electrical potential distributions within the cochlear volume conductor were modeled under time-dependent conditions based on the fundamental charge conservation Equation (1), using the dielectric parameters listed in Table 1.

(1) $$\nabla \cdot \left(\sigma \left(r\right)\nabla V\left(r,t\right)+\varepsilon \left(r\right)\nabla \frac{\partial V(r,t)}{\partial t}\right)=0$$

where σ(r) and ε(r) represent the spatial distributions of electric conductivity (Table 1) and relative permittivity across cochlear tissues, respectively, while V(r,t) is the dynamic electric potential field. This continuous transient formulation explicitly incorporates both resistive tissue conduction and capacitive displacement currents. The relative permittivity ($\epsilon_{r}$) of ST was set to $1\times 10^{3}$, while the relative permittivity values of the other tissue layers were selected based on the simulation frequency using the tissue properties from https://itis.swiss/virtual-population/tissue-properties/database/dielectric-properties (Accessed date: 12 June 2026).

**Table 1:** Tissue conductivities of cochlear structures used in FEM models of the cochlea.

Tissue Layer	Conductivity (S/m)	References
Scala tympani (ST)	$\sigma_{1}–\sigma_{6}$ (1.40, 1.45, 1.50, 1.55, 1.60, 1.65)	[29]
Cochlea wall	0.3	[30]
Basilar membrane	0.0125	[31]
Spiral ligament	1.67	[31]
Stria vascularis	0.0053	[31]
Spiral ganglion	0.33	[31]
Artery	0.32	[32]
Bone	0.0156	[30,31]
Silicone	$1\times 10^{-10}$	[31]

As illustrated in Figure 3, a sinusoidal voltage excitation with a peak-to-peak amplitude of $V_{\mathrm{in}}=1.0\;V_{p\text{-}p}$ at a operating frequency of f=1kHz was applied across the active measurement chain. The setup comprised a targeted bipolar electrode pair ($E_{i},E_{i-1}$) connected in series with a precision current-sensing resistor R (3.3 Ω). The instantaneous current flowing through the volume conductor $I_{\mathrm{el}}\left(t\right)$ was determined by monitoring the voltage drop across the sense resistor using Equation (2):

(2) $$I_{\mathrm{el}}\left(t\right)=\frac{V_{R}\left(t\right)}{R}$$

where $V_{R}\left(t\right)$ denotes the time-dependent voltage measured across the sensing resistor *R*. The transient impedance across the electrode pair $Z_{\mathrm{EP}}\left(t\right)$ was subsequently computed from the differential potential across the pair ($V_{\mathrm{EP}}\left(t\right)=V_{E_{i}}\left(t\right)-V_{E_{i-1}}\left(t\right)$) and the derived branch current. The electrode array was excited by a sinusoidal voltage source of 1V amplitude at $f_{0}=1\;\mathrm{kHz}$, connected in series with a sense resistor R=3.3Ω. Assuming a linear and time-invariant system operating in sinusoidal steady state, the impedance between the two electrodes, $Z_{EP}$, was defined as the ratio of the phasor of the inter-electrode voltage, ${\tilde{V}}_{EP}$, to the phasor of the electrode current, ${\tilde{I}}_{el}$, at $f_{0}$. The current phasor was obtained from the voltage across the sense resistor as ${\tilde{I}}_{el}={\tilde{V}}_{R}/R$, which yields Equation (3)

(3) $$Z_{EP}\left(j\omega_{0}\right)=\frac{{\tilde{V}}_{EP}}{{\tilde{I}}_{el}}=R\;\frac{{\tilde{V}}_{EP}}{{\tilde{V}}_{R}},\;\omega_{0}=2\pi f_{0},$$

where ${\tilde{V}}_{EP}$ and ${\tilde{V}}_{R}$ denote the complex phasors of the inter-electrode voltage and of the voltage across *R*, respectively. The magnitude and phase of the impedance follow directly from Equation (4):

(4) $$\left|Z_{EP}\right|=R\;\frac{\left|{\tilde{V}}_{EP}\right|}{\left|{\tilde{V}}_{R}\right|},\;∠Z_{EP}=∠{\tilde{V}}_{EP}-∠{\tilde{V}}_{R}.$$

Phasors ${\tilde{V}}_{EP}$ and ${\tilde{V}}_{R}$ were extracted from the simulated steady-state waveforms at $f_{0}$, and the impedance magnitude $|Z_{EP}|$ was used in all subsequent analyses.

Interfacial boundaries between distinct biological tissues were assumed to follow continuous potential and normal current density distributions:

(5) $$n\cdot \left(J_{1}-J_{2}\right)=0$$

where $J=\sigma E+\varepsilon \frac{\partial E}{\partial t}$ denotes the total current density vector, ensuring physically valid continuity across tissue interfaces.

To evaluate bioimpedance fluctuations during surgical array deployment, the electrode carrier was systematically translated along a predefined 25mm trajectory. At each discrete insertion coordinate, a full transient solver pass was executed, enabling the extraction of time-resolved bioimpedance dynamics across all defined electrode pairs as a function of insertion depth, array orientation, and local tissue proximity. The impedance measurements were performed directly through the implanted multi-electrode array; no separate probe was used. For each of the eight pairs of bipolar electrodes, the potential difference was measured using Equation (1), and the impedance was subsequently calculated in post-processing using Equation (4).

## 3. Results

### 3.1. Complex Electrical Parameters and Impedance Characterization

**Figure 5:**
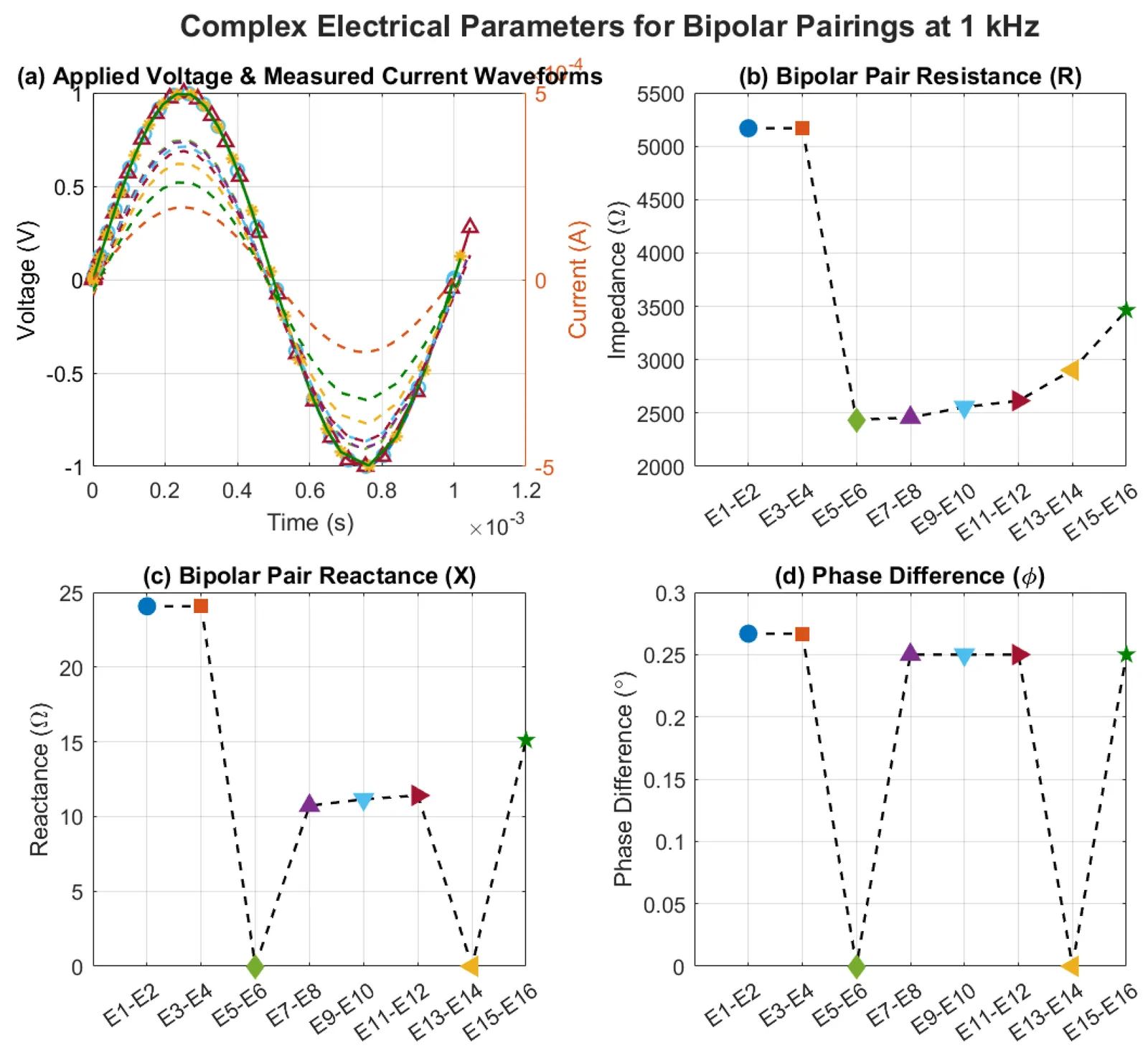
Effect of complex impedance on the electrical response. (**a**) Induced electrical potential across each electrode pair (left *y*-axis) and measured circuit current (right *y*-axis). (**b**) Magnitude of the bipolar electrode-pair impedance as a function of electrode pair. (**c**) Magnitude of the bipolar electrode-pair reactance as a function of electrode pair. (**d**) Phase difference between the applied voltage and the calculated current. Results for each electrode pair are highlighted in different colors.

To characterize the underlying bioelectric response across the bipolar electrode channels (E1–E2 through E15–E16), the complex impedance was evaluated at an operating frequency of 1kHz based on middle electrode insertion. The analysis included resistance (*R*), reactance (*X*), phase angle (ϕ), and transient current–voltage response as shown in Figure 5. As illustrated in Figure 5a, application of a 1V sinusoidal excitation resulted in stable and approximately proportional current responses across all eight bipolar electrode pairs. The peak current amplitudes ranged from approximately $1.5\times 10^{-4}\;A$ to $4.0\times 10^{-4}\;A$.

The calculated *R* variation based on complex impedance characterizartion is shown in Figure 5b. The derived *R* exhibits a distinct spatial variation across the electrode pairs. The first two channels, E1–E2 and E3–E4, showed relatively stable resistance values, remaining close to 5100 Ω. A pronounced reduction in *R* is observed at E5–E6. Following this decrease, *R* increased progressively across the subsequent electrode pairs, extending from E5–E6 to E15–E16. This systematic variation in resistance indicates that the electrical properties of the measured bioelectric pathway are not spatially uniform and may reflect differences in the underlying tissue or electrode–tissue interface characteristics. The calculated *R* variation based on complex impedance characterizartion is shown in Figure 5b. The derived *R* exhibits a distinct spatial variation across the electrode pairs. The first two channels, E1–E2 and E3–E4, showed relatively stable resistance values, remaining close to 5100 Ω. A pronounced reduction in *R* is observed at E5–E6. Following this decrease, the *R* increased progressively across the subsequent electrode pairs, extending from E5–E6 to E15–E16. This systematic variation in resistance indicates that the electrical properties of the measured bioelectric pathway are not spatially uniform and may reflect differences in the underlying tissue or electrode–tissue interface characteristics.

The results for *X* and ϕ based on full electrode insertion are shown Figure 5c,d, respectively. The *X* and ϕ shows the same variation with *R* for initial three electrode pairs from E1–E2 and E5–E6. However, the *X* and ϕ and ϕ exhibits similar trend for all electrode pairs. The pairs E1–E2 and E3–E4 generated the largest reactive response at approximately 24.1Ω. This is valid for the ϕ variations as well. These results confirm that while total impedance magnitude across bipolar pairings is overwhelmingly dominated by the resistive component (R≫X), spatial positioning introduces subtle localized phase shifts and reactive variations along the array length.

### 3.2. Sensitivity Analysis of Insertion Impedance to Conductivity Range

**Figure 6:**
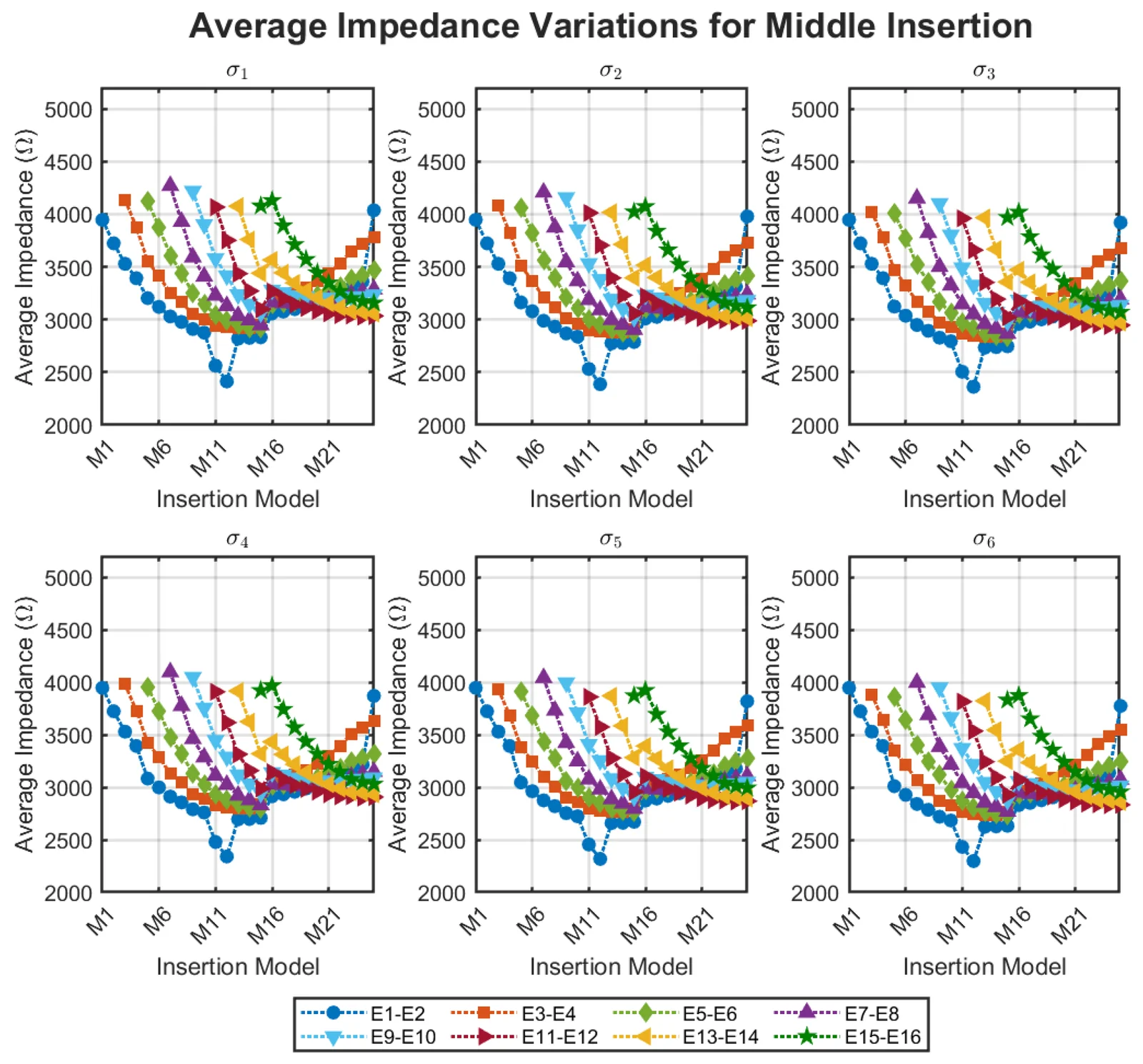
Average impedance variations (Ω) across sequential insertion models (M1–M25) for middle insertion under six conductivity conditions ($\sigma_{1}$ through $\sigma_{6}$). Results for each electrode pair are highlighted in different colors.

To evaluate the influence of spatial positioning on electrical behavior, average impedance profiles were tracked across progressive insertion steps (M1–M25) for eight bipolar electrode configurations E1–E2 through E15–E16 under six conductivity conditions ($\sigma_{1}$–$\sigma_{6}$). The results for middle insertion trajectory presents a highly consistent V-shaped profile followed by a secondary rebound as shown in Figure 6. Initial entry values (M1) ranged between 4000Ω and 4300Ω, declining rapidly to a prominent minimum at M11–M13 (2300–2500Ω for the primary pair E1–E2, and 2800–3000Ω for higher-order pairs). Subsequent insertion produced a secondary elevation peaking near M15–M16 (≈3200 Ω) before plateauing between 2900Ω and 3300Ω through M25. This biphasic pattern remained remarkably stable across all tested conductivity states ($\sigma_{1}–\sigma_{6}$), indicating that the geometric trajectory dominates over environmental conductivity in determining impedance trends during middle insertion.

**Figure 7:**
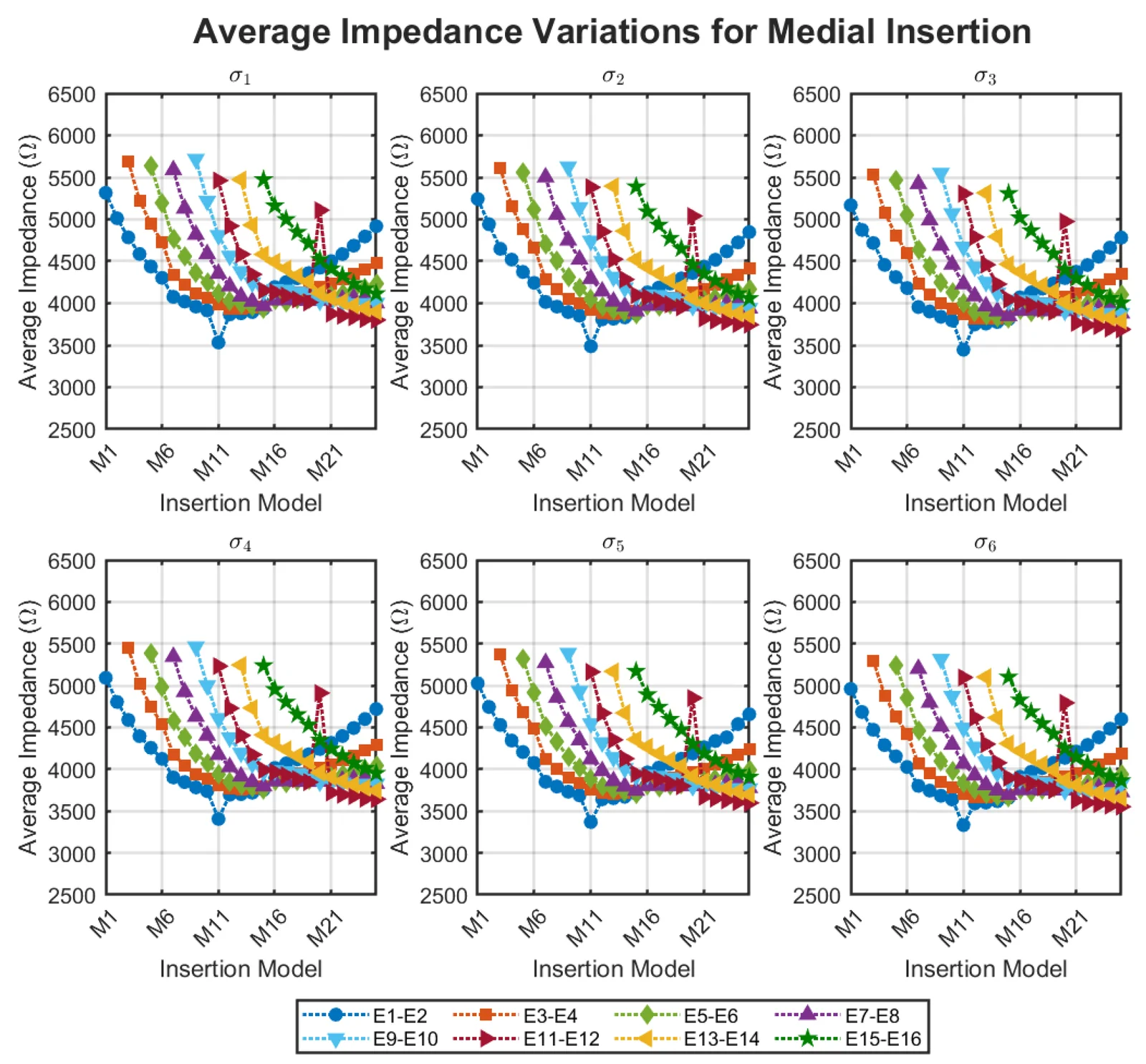
Average impedance variations (Ω) across sequential insertion models (M1–M25) for medial insertion under six conductivity conditions ($\sigma_{1}$ through $\sigma_{6}$). Results for each electrode pair are highlighted in different colors.

Figure 7 shows medial insertion results. Initial measurements across all conductivity environments exhibited starting impedance values near 5000–5700Ω for peripheral pairings, followed by a monotonic decline toward local minima around models M11–M13 (≈3300–3750 Ω). While most channels stabilized at baseline levels past M15, deeper pairings such as E11–E12 displayed localized transient peaks reaching upwards of 5000Ω near M19–M20, highlighting region-specific impedance sensitivity during medial advancement.

**Figure 8:**
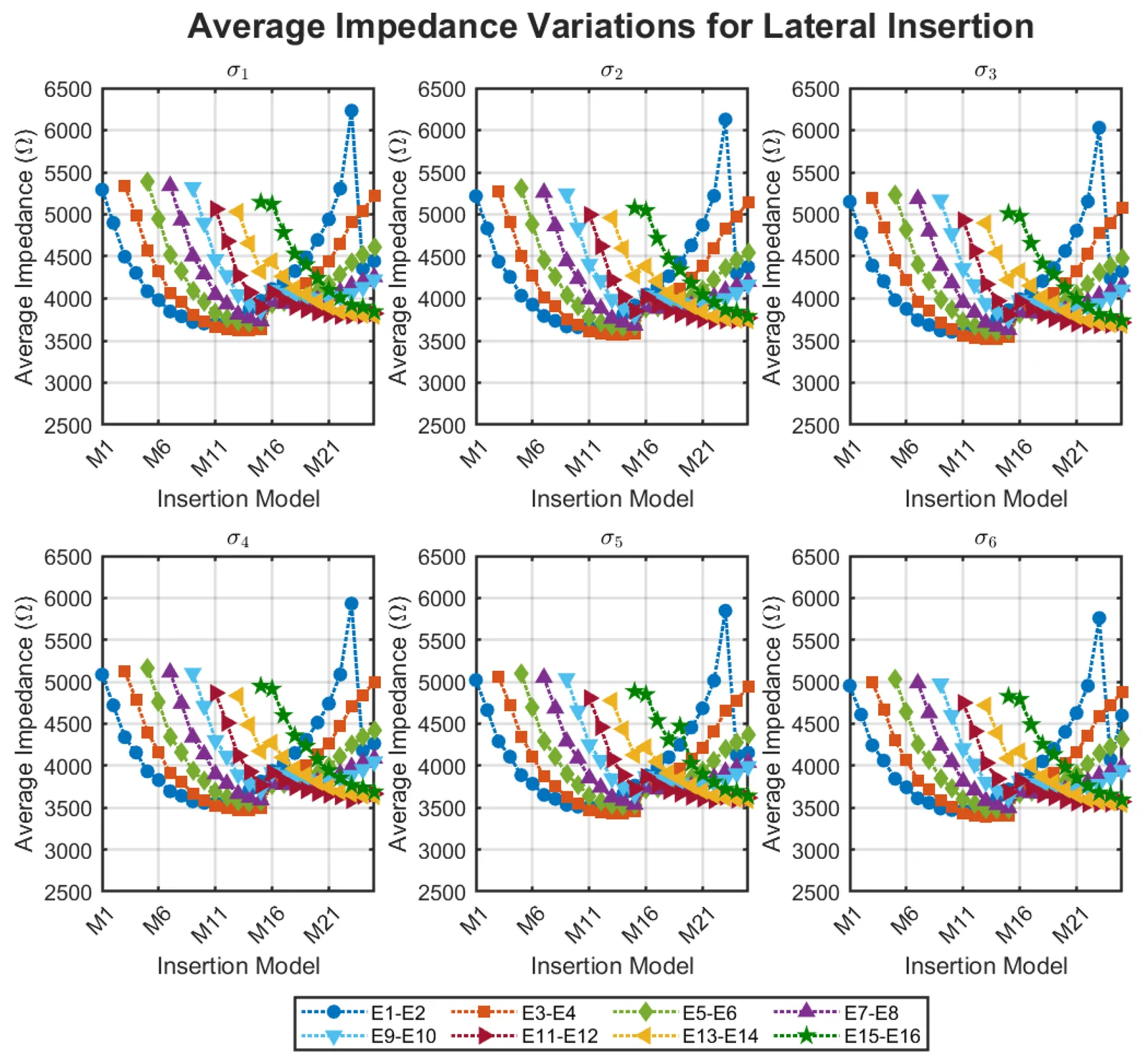
Average impedance variations (Ω) across sequential insertion models (M1–M25) for lateral insertion under six conductivity conditions ($\sigma_{1}$ through $\sigma_{6}$). Results for each electrode pair are highlighted in different colors.

The result for the lateral insertion is shown in Figure 8. The lateral insertion profile demonstrated a distinctly sharp divergence in terminal behavior compared to the medial and middle paths. Although the initial insertion steps (M1–M13) mirrored the typical parabolic decay—reaching local minima near 3400–3600Ω—the apical pairing (E1–E2) exhibited a steep exponential increase in impedance beyond M16, rising rapidly to terminal peaks approaching 4500–6200 Ω at M24–M25. Remaining bipolar pairs (E3–E4 through E15–E16) maintained moderate, steady-state profiles (≈3500–4300 Ω), confirming that lateral trajectory constraints specifically elevate apical channel impedance in deep insertion phases. Across all three trajectories, variations in medium conductivity ($\sigma_{1}–\sigma_{6}$) altered absolute magnitude only marginally while preserving channel-specific morphology and inflection points.

### 3.3. Impedance Profiles Relative to Middle Insertion

**Figure 9:**
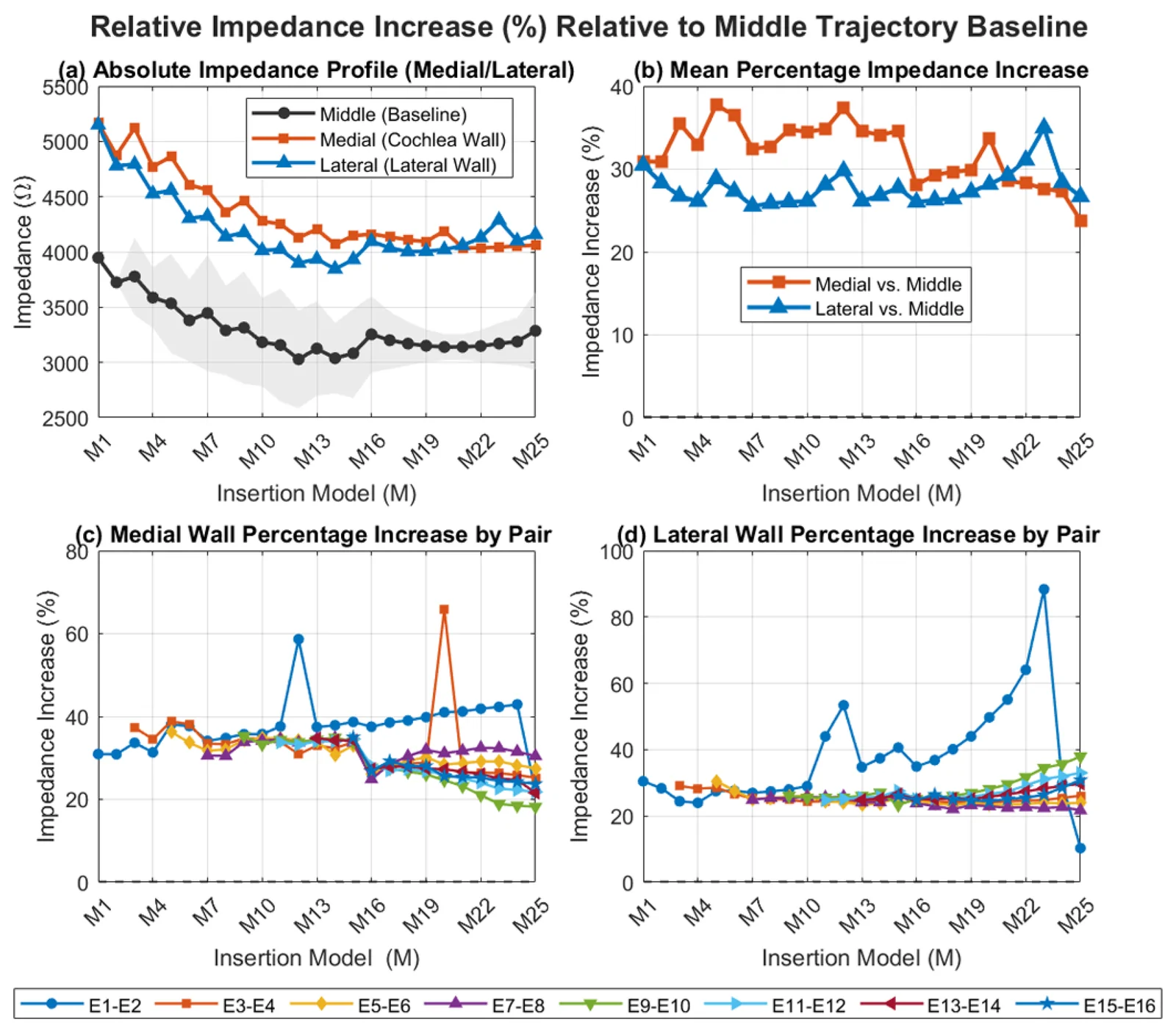
Relativefirm that the correct form was used in the figure. Any apparent discrepancy may be due to the figure size. percentage increase in impedance caused by wall proximity during insertion. (**a**) Absolute impedance trajectories (Mean±SD) comparing middle insertion baseline against medial and lateral trajectories. (**b**) Mean percentage impedance increase (%ΔZ) for medial vs. middle and lateral vs. middle trajectories across 25 insertion steps (M1–M25). (**c**) Pair-wise percentage impedance increase along the array for medial wall proximity. (**d**) Pair-wise percentage impedance increase along the array for lateral wall proximity relative to the center baseline.

To investigate the effect of intra-cochlear electrode position on impedance characteristics, the electrical responses obtained along the medial trajectory (cochlear wall) and lateral trajectory (lateral wall) were systematically compared with those measured along the central trajectory as shown in Figure 9, which served as the reference condition, for insertion models M1–M25. Figure 9a compares the baseline middle trajectory against the medial and lateral configurations. Across all insertion depth models (M1–M25), both wall-adjacent trajectories consistently exhibited higher absolute impedance magnitudes than the baseline trajectory. While the middle baseline decreased smoothly from approximately 3950 Ω at M1 to 3000–3300 Ω in deeper models, proximity to either boundary elevated overall impedance values above 4000 Ω across the full insertion progression.

Figure 9b presents the mean relative percentage increase in impedance compared to the middle baseline across all bipolar pairs. The results demonstrate distinct spatial trends for the two boundary trajectories. For the medial trajectory, the impedance increased by approximately 24–38% relative to the middle reference. The largest deviations occurred during the early-to-intermediate insertion stages, particularly between M2 and M13. Peak increases of approximately 38% occurred at M5 and M12, whereas the relative increase decreased to approximately 24% at the deepest insertion position, M25. The lateral trajectory exhibits a comparable but somewhat different pattern, with mean impedance increases ranging from approximately 25% to 35%. In this case, the largest deviations occurred at the beginning and near the end of the insertion sequence, with increases of approximately 31% at M1 and 35% at M23. Between M6 and M20, the impedance increase remained comparatively stable, generally within the 26–29% range.

Figure 9c and Figure 9d outline the pair-by-pair relative percentage increases for medial and lateral configurations, respectively.

Analysis at the individual bipolar-channel level further demonstrated that impedance sensitivity varied according to electrode position and proximity to the cochlear boundaries. For the medial trajectory, most channels, from E3–E4 through E15–E16, exhibited relatively consistent impedance elevations ranging from approximately 18% to 38%. Nevertheless, notable localized increases were observed in the leading electrode pairs. The E1–E2 pair showed a pronounced transient response, with the impedance increase reaching approximately 59% at M12. Similarly, the E3–E4 pair exhibited a sharp increase at M20, reaching approximately 65%. A stronger spatial dependence was observed for the lateral trajectory, particularly for the E1–E2 pair. This channel initially exhibited an impedance increase of approximately 25–30%, followed by a secondary maximum of approximately 54% at M11. A substantial increase then developed from approximately M18, ultimately reaching an 88% elevation at M23. In comparison, the remaining bipolar electrode pairs along the lateral trajectory demonstrated considerably more stable behavior, with their relative impedance increases generally confined to the 20–38% range throughout the insertion sequence. These findings indicate that electrode proximity to the cochlear boundaries can substantially influence impedance magnitude, with certain electrode pairs exhibiting markedly greater spatial sensitivity than others.

**Figure 10:**
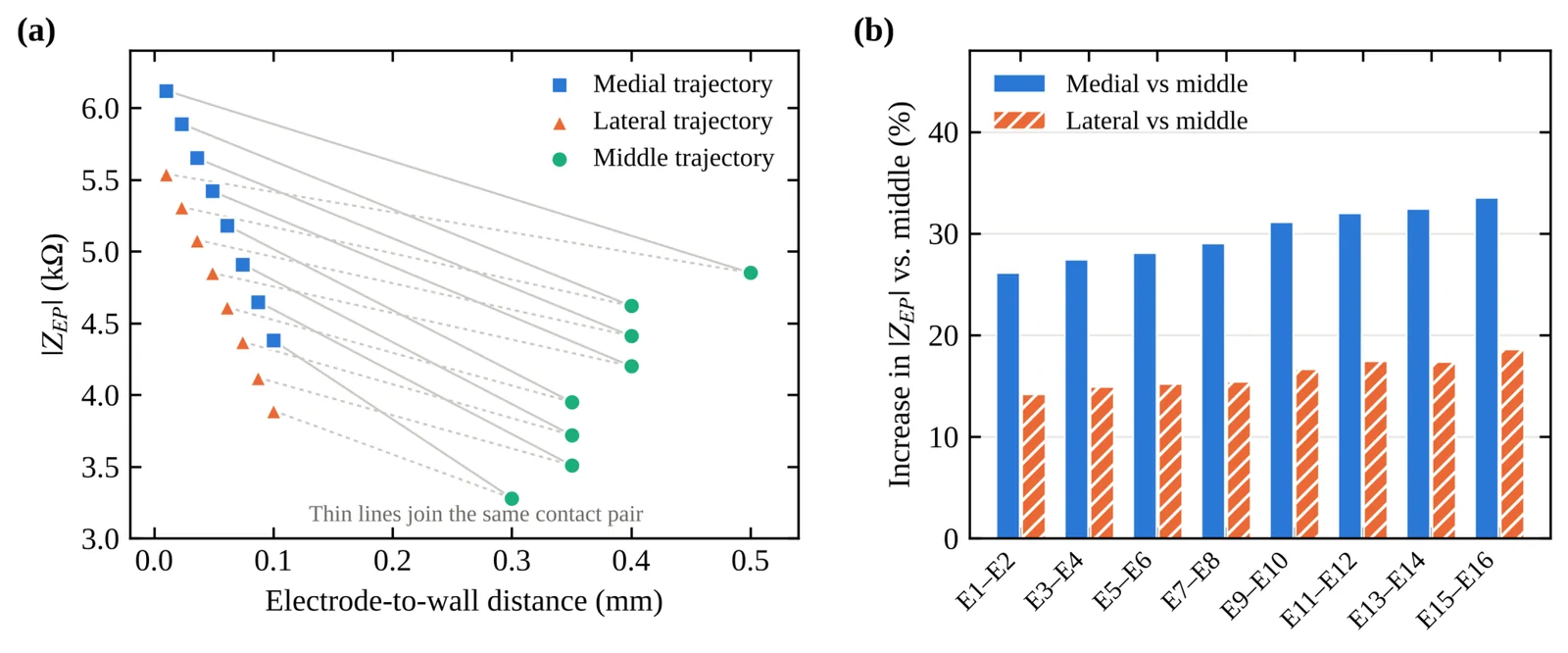
Relationship between impedance and electrode-to-wall distance. (**a**) Impedance magnitude $|Z_{EP}|$ of each contact pair as a function of its distance to the nearest cochlear wall for the medial, lateral and middle trajectories; thin lines connect the same contact pair across trajectories. (**b**) Increase in $|Z_{EP}|$ of the medial and lateral trajectories relative to the middle trajectory for each contact pair.

To quantify the relationship between impedance and electrode-to-wall distance, the shortest distance between each contact pair and the nearest cochlear wall was extracted from the model geometry for all three trajectories, and $|Z_{EP}|$ was plotted against this distance (Figure 10a). As shown in Figure 10a, the impedance of all trajectories decreased by approximately 1.6–1.7 kΩ from E1–E2 to E15–E16, including along the middle trajectory, where the wall distance itself decreased from 0.5 to 0.3 mm. This longitudinal trend therefore reflects the position of each pair along the cochlea rather than its proximity to the wall. The effect of wall proximity was assessed by comparing the trajectories at the same contact pair, using the middle trajectory (0.30–0.50 mm from the wall) as the reference (Figure 10b). Reducing the distance to 0.01–0.10 mm increased $|Z_{EP}|$ by 610–690 Ω (14.2–18.6%) at the lateral wall and by 1100–1270 Ω (26.2–33.5%) at the medial wall, and this increase was consistent across all eight contact pairs (Figure 10b). A linear regression with pair-specific intercepts showed that $|Z_{EP}|$ decreased by 188±25Ω per 0.1 mm increase in electrode-to-wall distance (95% confidence interval, $R^{2}=0.99$), with an additional 574±84Ω when the array approached the medial rather than the lateral wall. These results indicate that wall proximity can be inferred from the change in impedance relative to a pair-specific reference, but not from the absolute impedance value alone, and that the medial and lateral walls produce distinguishable impedance signatures.

## 4. Discussion and Conclusions

This study investigated the complex relationships between electrode–ground and bipolar channel impedance, electrode proximity to surrounding intra-cochlear structures, and insertion depth using detailed FEM simulations. Utilizing a high-resolution, multilayered 3D anatomical volume conductor model of the human cochlea constructed from μCT datasets, an electrode array based on the Oticon EA with 20 platinum–iridium electrodes was integrated into the ST geometry. By evaluating spatial impedance dynamics across 16 sequential electrode pairings (E1–E2 through E15–E16), insertion positions (M1–M25), and diverse environmental conductivity conditions ($\sigma_{1}–\sigma_{6}$), this framework provides a biophysical foundation for real-time trajectory guidance.

At first, the impact of the complex impedance on the overall system outcome was investigated for a fully inserted electrode array (EA). As shown in Figure 5, the overall reactance magnitude remained negligible compared to the total resistance (X<25Ω versus R>2400Ω), confirming that capacitive reactance contributes less than 0.5% to the total bipolar impedance magnitude. This minor capacitive contribution may be correlated with the relatively lower excitation frequency used in this study, consistent with the previous literature indicating that the capacitive effects of biological tissue become significant only at higher frequencies (e.g., above 10kHz) [33,34].

Computational analysis across Figure 6, Figure 7 and Figure 8 demonstrates a clear inverse relationship between local tissue conductivity ($\sigma_{1}–\sigma_{6}$) and electrical impedance across all insertion pathways. Increasing conductivity monotonically reduces absolute impedance due to lower ohmic resistance in the perilymph. However, proximity to non-conductive anatomical boundaries (medial and lateral) significantly heightens sensitivity to these shifts by constraining current lines and altering the scalar potential field. This behavior aligns with cochlear electrodynamics under the quasi-static approximation. With negligible capacitive/inductive phase shifts (ϕ≈0°), the potential distribution is governed by Laplace’s equation (∇·(σ∇Φ)=0). While conductivity variations ($\sigma_{1}–\sigma_{6}$) scale absolute impedance magnitudes, they do not alter global curve morphology. Instead, spatial array trajectory and boundary proximity dominate profile shape.

As detailed in Table 2, boundary proximity produces substantial relative impedance shifts compared to the middle trajectory baseline. Mean percentage increases ranged from 24–38% for medial insertions and 25–35% for lateral insertions. During initial stages (M1–M5), medial alignment induced a 31–38% elevation while lateral placement yielded 26–30%. At terminal depth (M25), these shifts converged to approximately 24% (medial) and 27% (lateral). All pathways exhibited a non-monotonic “U-shaped” impedance evolution. Values initially dropped during early insertion (M1–M13, ≈2300–3000 Ω) as electrode immersion expanded contact area within the wide central lumen. In deeper insertion (M16–M25), trajectories diverged dramatically due to localized wall contact. Along the medial boundary, prominent transient spikes occurred at pair E1–E2 (≈59% at M12) and E3–E4 (≈65% at M20). Conversely, the lateral pathway induced extreme localized sensitivity at the lead pair E1–E2, peaking first at 54% (M11) and culminating in an 88% spike at M23. Secondary pair deviations remained bounded between 18–38% (medial) and 20–38% (lateral). Differential profiles (ΔImpedance) confirm that physical boundary proximity exerts a primary governing influence over channel impedance, far outweighing baseline conductivity shifts.

The impedance variations based on three insertion profiles showed U-shaped impedance dynamics—dropping near the ST center followed by a terminal rise—as shown in Figure 6, Figure 7 and Figure 8. Within the simulated conditions, these findings indicate that multi-channel electrical impedance is sensitive to the proximity of the electrode array to the cochlear boundaries. This sensitivity suggests a possible role for impedance monitoring in anticipating wall contact and in guiding trajectory adjustments during automated cochlear implant insertion, although such applications have not been tested in the present work. The characteristic U-shaped impedance profile and its dependence on boundary proximity could, if confirmed experimentally, serve as a source of intraoperative feedback for clinicians. This is relevant because intracochlear trauma, including perforation of the basilar membrane, damage to the osseous spiral lamina and translocation of the array into the scala vestibuli, is largely caused by contact forces along the cochlear walls that the surgeon cannot perceive directly [31]. As the inside of the cochlea cannot be visualised during surgery, manual insertion offers only limited sensory feedback, and the forces that precede structural damage often go unnoticed [5]. Detecting the shift of the array from the low-impedance central region of the scala towards the higher-impedance wall regions could therefore complement speed-controlled robotic insertion systems. In such a setting, impedance signals monitored by the surgeon or supplied to a robotic insertion device might allow the trajectory to be adjusted before irreversible damage occurs. Whether impedance measurements can provide this information reliably and quickly enough in practice, however, must first be established in cadaveric temporal bone experiments and subsequently in vivo.

Several practical and translational limitations should be considered when interpreting these findings. Although the 3D FEM model incorporates realistic μCT-derived geometries and multilayered tissue properties, clinical in vivo insertions involve dynamic mechanical deformation, complex bony structures, and biological membranes that may introduce additional nonlinear variations not captured by the current model [35]. Experimental validation using physical cochlear models, ex vivo specimens, or intraoperative measurements would further strengthen the interpretation of the observed impedance trends.

Clinical translation of the proposed approach depends on whether the impedance variation in commercial cochlear implants offers sufficient resolution. Recent clinical work has shown that this can detect when individual contacts enter the cochlea during insertion [17]; however, this relies on the large impedance increase between air and cochlear fluid, with all contacts updated only every one to two seconds. The impedance magnitudes obtained in the present study lie within the range reported in clinical intracochlear measurements [17], which supports the plausibility of the model. Nevertheless, these values were derived from only three insertion trajectories in a single cochlear geometry, and a more extensive investigation covering a wider range of insertion conditions and cochlear anatomies is needed to confirm them.

The clinical use of the proposed approach raises the question of whether the patient could perceive the sensing signal. If the amplitude of the 1kHz intracochlear current exceeds the threshold for electrical hearing, it may activate the auditory nerve and evoke a sound sensation. Since most cochlear implantations are performed under general anesthesia, this would not be experienced consciously in the majority of cases, and impedance testing through intracochlear contacts is already part of routine surgical practice. For patients operated on under local anesthesia, the sensing current can be limited to subthreshold levels, as neural activation is not needed for impedance estimation, and higher excitation frequencies can further reduce the likelihood of nerve activation. Future experimental studies will be needed to determine an excitation amplitude and frequency that provide reliable impedance measurements while avoiding neural stimulation and maintaining electrochemical safety.

The present findings are derived entirely from computational modelling, and their accuracy therefore depends on the assumed geometry, tissue conductivities and boundary conditions. Although the model is based on an anatomically realistic reconstruction of the human cochlea and uses tissue properties reported in the literature, it does not capture all sources of variability encountered in vivo, such as inter-subject differences in cochlear anatomy, the electrode–tissue interface, tissue trauma and fluid displacement during insertion. Experimental validation is therefore an essential next step. Future work will compare the simulated impedance profiles with impedance measurements recorded during insertion in human cadaveric temporal bones, with the corresponding electrode position confirmed by post-insertion imaging and complemented by insertion-force measurements. These experiments will also establish whether the characteristic impedance patterns identified here can be related quantitatively to specific electrode–wall distances and insertion trajectories, which would provide an objective electrical measure of electrode placement. Finally, bringing continuous, contact-specific impedance feedback into the operating theatre will require dedicated measurement hardware and possibly changes to implant or monitoring-system firmware, and clinically relevant safety thresholds will need to be defined for both surgeon-guided and robot-assisted insertion.

## Figures and Tables

**Table 2:** Quantitative summary of relative impedance changes by trajectory.

Metric/Metric Range	Medial vs. Middle	Lateral vs. Middle
Mean percentage increase (%)	24–38%	25–35%
Initial stage increase (M1–M5)	≈31–38%	≈26–30%
Terminal stage increase (M25)	≈24%	≈27%
Prominent channel spikes	E1–E2 (≈59% at M12)	E1–E2 (≈54% at M11)
	E3–E4 (≈65% at M20)	E1–E2 (≈88% at M23)
Secondary pair deviations	18–38%	20–38%

## Data Availability

The data supporting the findings of this study are available from the corresponding author upon reasonable request.
